# Surgical Approach to Anorectal Melanoma with PET-CT Staging: A Case Report

**DOI:** 10.1055/s-0037-1608902

**Published:** 2017-11-27

**Authors:** Nilufer Bulut, Sevinc Dagıstanlı, Burcak Yılmaz, O. Faruk Atay

**Affiliations:** 1 Department of Medical Oncology, Kanuni Sultan Suleyman Search and Education Hospital, Istanbul, Turkey; 2 Department of General Surgery, Kanuni Sultan Suleyman Search and Education Hospital, Istanbul, Turkey; 3 Division of Nuclear Medicine, Department of Radiology, Sisli Etfal Education and Research Hospital, Istanbul, Turkey; 4 Department of Pathology, Kanuni SS Search and Education Hospital, Istanbul, Turkey

**Keywords:** anorectal melanoma, PET-CT, abdominoperineal resection

## Abstract

Rectal hemorrhage should be evaluated within a wide spectrum ranging from benign diseases to a malignant process. Especially, the melanomas of rectum are detected at an advanced stage when diagnosed since the present symptoms of rectal melanomas are similar. The question of what will be the surgical approach with MR, CT, and PET-CT imaging methods performed after histopathological diagnosis still conserves its topicality. PET-CT is a good imaging method for determination of distant metastasis and lymphatic involvement. In the present case, a patient with early-stage rectal melanoma was treated with APR. No relapse/metastasis was detected during the 18-month follow-up. The aggressive course of the disease and its low response rates to medical treatments may cause the surgical approaches to be more extensive.

Anorectal melanomas are infrequent cancers accounting for 0.05 to 2% of all colorectal cancers. 1 Due to lack of a specific symptom, this type of cancer can be misdiagnosed as a benign lesion of colorectal in patients with rectal bleeding. Conventional treatment modality is a surgery that involves abdominoperineal resection (APR) or extensive surgical excision. 1 Although advanced staging techniques suggest a course of action in the preoperative period, the potential of frequent relapse and lack of a standard treatment approach currently forces the surgeon to the preference of a more aggressive operation chain.

The 18F-fludeoxyglucose (FDG) PET/CT is an up-to-date approach in determining lymph node involvement and distant metastasis at the same time. The preferred surgical approach and lymph nodes involved are indicative for determining disease-specific survival.

## Case Report

**Fig. 1:**
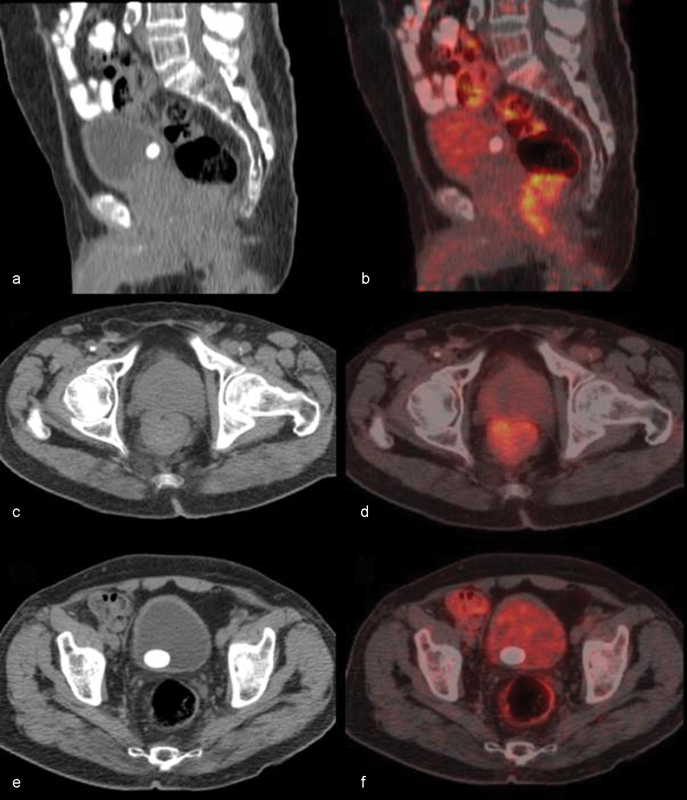
The F-18 FDG-PET/CT revealed primary rectal tumoral lesion, and regional lymph nodes were FDG (−). (a) Sagital CT, (b) Sagital fusion, (c) Axial CT and (d) Axial fusion images of the primary hipermetabolic rectal malign melanoma (e) Axial CT and (f) Axial fusion images of milimetric FDG(−) pelvic lymph nodes.

**Fig. 2:**
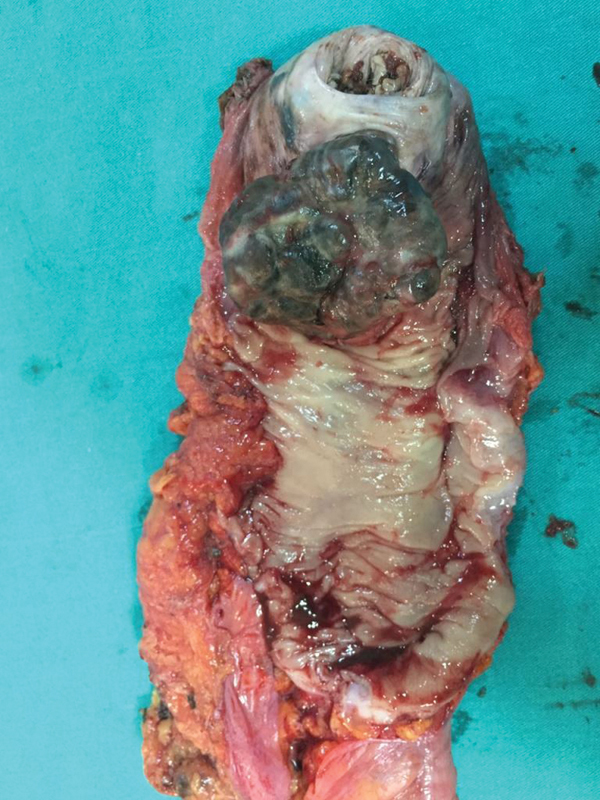
Gross findings of the resected specimen. Polypoid blackish tumor was observed at 2 cm proximal to the dentate line.

**Fig. 3:**
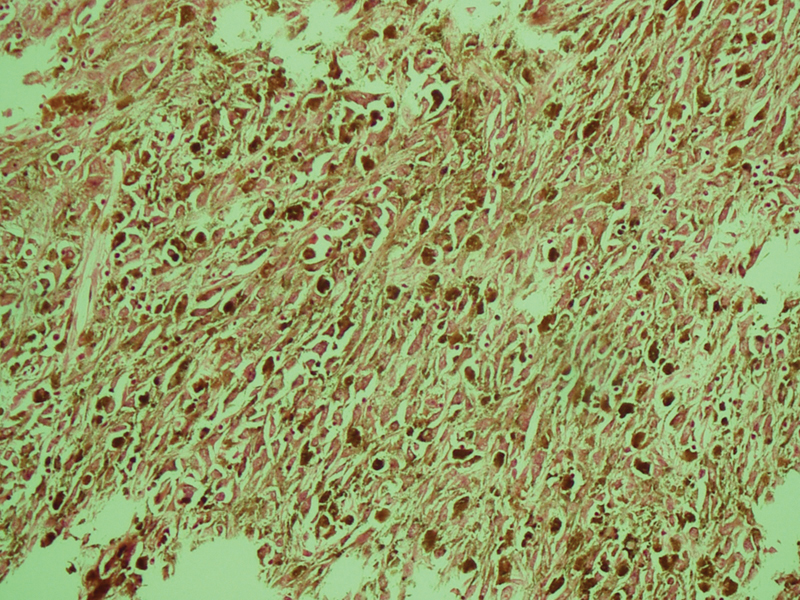
The S-100 and hematoxylin eosin staining showed increased mitosis and increased number of nucleoli. There were pleomorphic cells containing melanin pigment.

A 72 year-old male patient presented with rectal bleeding and generalized weakness. Colonoscopy revealed a 5-cm ulcerovegetan mass lesion on the posterior wall of the lower part of rectum. There were diverticular openings in the sigmoid and descending colon. Inguinal lymph nodes were not palpable on clinical examination. Hemoglobin was 8 g/dL, carcinoembryonic antigen (CEA) was 3 ng/mL, and human immunodeficiency virus (HIV) test was negative. The biopsy specimen underwent histopathological examination and the diagnosis of a melanoma was established with immuno-staining HBM-45 (+), CK7 (−), CK20 (−). No lymph nodes were detected on abdominal computed tomography (CT) for staging purposes. Positron emission tomography-CT (PET-CT) revealed an increased hypermetabolic density in the rectal area with intraluminal protrusion and 9-mm lymph nodes in the right internal iliac, right common iliac, aortocaval, left paraaortic areas with minimal metabolism. There were reactive lymph nodes with hilar fat measuring 22 mm at most without and FDG uptake in the bilateral inguinal fossae ( Fig. 1 ). The patient underwent APR due to lack of distant metastasis. In the rectum specimen, a 6 × 5 cm black vegetan mass lesion was observed invading the anal sphincter and involving a 2-cm segment above the sphincter ( Fig. 2 ). The pathological examination revealed the tumor tissue in the internal sphincter. The radial and upper resection margins were intact. Reactive hyperplasia was observed in the lymph nodes. The S-100 and hematoxylin eosin staining showed an increased mitosis and increased number of nucleoli. There were pleomorphic cells containing melanin pigment ( Fig. 3 ). The result was reported as stage 1 spindle-cell malignant melanoma with polypoid appearance (Slingluff's clinical stage). 2

## Discussion

Anorectal melanomas arise from the dentate line of which 65% are located in the anal canal or anal wedge. 3 These lesions are located in the first 6 cm in the anal canal. The most common symptoms are rectal bleeding, pain, altered bowel habits, and prolapse of the mass. The lesions are often polypoid and hyperpigmented and they may sometime become ulcerated, while 30% of the lesions are amelanotic. They may present with an epithelioid, spindle cell, lymphoma-like and pleomorphic type. 1 Anal melanomas are often associated with the involvement of inguinal lymph nodes, distant metastasis, and synchronous and metachronous adenocarcinomas. Tumor volume and presence of obstructive findings are indications for surgery and APR is often preferred in such cases. 4 The local relapse is especially frequent in tumors with the volume cutoff value of ≥ 3.5 cm. Whereas lymph node involvement is higher in those measuring > 6 cm and their rates of sphincter preservation is lower. 5 Compared with extensive surgical excision, APR offers favorable local management; however, it does not improve overall survival. 6 7 In the literature, there are several but limited data showing a contribution to survival in long-term follow-up. Tumor biology, presence of perineural invasion, and lymphovascular involvement are the main determinants of prognosis. In particular, PET-CT is the most commonly used method in delineating lymphatic involvement. 8

A wider surgical approach was preferred in the present case due to the malignant characteristic of the tumor, lymph node involvement in the PET-CT, and its localization in the lower rectum. The advanced age of the patient would reduce his tolerance to reexcision and response to radiotherapy. 5 It was considered that local control would be better achieved with APR. Besides, the detection of tumor tissue in the internal sphincter showed that a correct surgical preference was made. No relapse was detected during the 18-month follow-up of the patient.

Today, the preference of APR or wide local excision was investigated in numerous studies, and no difference was detected in 5-year survival analysis, whereas local relapse rates were found to be 15.6% for APR and 64.7% for wide excision. 2 Abdominoperineal resection provides an improved local management, whereas adjuvant chemotherapy is not effective and the disease is often resistant to radiation therapy. 5

The comorbidity status of patient, postoperative complications, and permanent colostomy status caused disadvantages for APR, while the tumor volume over 5 cm, lymphovascular invasion, presence of lymph node involvement, distance to the anal surgical wedge, and concerns about providing safe surgical margin keeps the APR on the agenda for the surgeon.

## Conclusion

Malignant melanomas are rare aggressive tumors of the rectum. Currently, PET-CT is the most widely adopted modality in visualizing perirectal lymph nodes and screening for distant metastasis to evaluate the patient status for the options of curative surgery. Today, surgical approaches are still controversial. The histopathologic characteristics and stage of tumor, overall surveillance, and prediction of quality of life are the factors determining the preferences of the surgeon.
